# Surface Chelation Enabled by Polymer-Doping for Self-Healable Perovskite Solar Cells

**DOI:** 10.3390/nano12183125

**Published:** 2022-09-09

**Authors:** Kuiyuan Zhang, Xiangrong Shi, Guangyu Wu, Yudong Huang

**Affiliations:** 1 MIIT Key Laboratory of Critical Materials Technology for New Energy Conversion and Storage, School of Chemistry and Chemical Engineering, Harbin Institute of Technology, Harbin 150001, China; 2 School of Petrochemical Engineering, Changzhou University, Changzhou 213000, China; 3 College of Biology and the Environment, Co-Innovation Center for the Sustainable Forestry in Southern China, Nanjing Forestry University, Nanjing 210037, China

**Keywords:** self-healing, perovskite solar cell, ambient environment, high efficiency, energy harvesting

## Abstract

Polymer doping is an efficient approach to achieve self-healing perovskite solar cells. However, achieving high self-healing efficiency under moderate conditions remains challenging. Herein, an innovative self-healable polysiloxane (PAT) containing plenty of thiourea hydrogen bonds was designed and introduced into perovskite films. Abundant thiourea hydrogen bonds in PAT facilitated the self-healing of cracks at grain boundaries for damaged SPSCs. Importantly, the doped SPSCs demonstrated a champion efficiency of 19.58% with little hysteresis, almost rivalling those achieved in control atmosphere. Additionally, owing to the effective chelation by PAT and good level of thiourea hydrogen bonds, after 800 cycles of stretching, releasing and self-healing, the doped SPSCs retained 85% of their original IPCE. The self-healing characteristics were demonstrated in situ after stretching at 20% strain for 200 cycles. This strategy of pyridine-based supramolecular doping in SPSCs paves a promising way for achieving efficient and self-healable crystalline semiconductors.

## 1. Introduction

Stretchable hybrid organic–inorganic halide perovskite-based solar cells (SPSCs) are attractive photoelectric materials exhibiting the advantages of extraordinary power-conversion efficiencies (PCE over 18%), low cost, and easy manufacturing while exhibiting strong panchromatic sunlight absorption, long carrier diffusion lengths, and adjustable direct bandgaps [1,2,3]. SPSCs should attain high photovoltaic levels and properties fabricated by grid-connection PSCs, while synchronously sustaining remarkable stretchability and fatigue resistance [4,5,6]. However, poor crystallinity and fragility upon stretching would generate plenty of cracks at grain boundaries (GBs), consequently exacerbating the photovoltaic properties of SPSCs [7,8,9,10]. Nonradiative charge recombination, resulting from these defective GBs, would lead to the loss of photovoltaic efficiency and environmental stability in air [11,12]. Furthermore, cracks at GBs would rapidly spread to the whole device and the mechanical stability will become aggravated dramatically. Challengingly, the damaged areas struggle to heal themselves owing to the intrinsic brittleness of perovskite crystals [13,14]. In this context, various approaches on interface engineering and polymer doping have emerged to enhance the perovskite crystallinity of stretchable perovskite devices [14,15,16,17]. By doping organic molecules or metal oxide materials into films, the crystallinity could be enhanced prominently, and high stretchability (20% strain) can also be achieved [13,18,19]. On the other hand, PCE of SPSCs could also be improved with a satisfactory stretchability by doping into the polymer scaffolds interface of GBs [6]. However, self-healing is still the “Achilles’ heel” of optoelectronic and mechanical stretchability [13,20].

**Figure 1:**
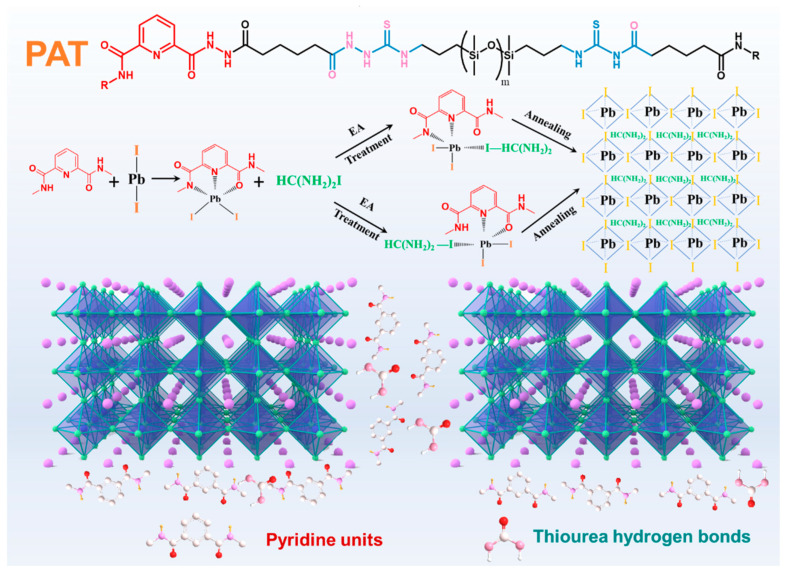
Proposed mechanisms of passivation and self-healing characteristics, and the physical structure of devices.

In our previous work, pyridine-based polysiloxane was prepared and introduced into perovskite films to enhance the crystallinity. However, the self-healing time and efficiency still need to be improved, owing to the low transferability of urea-based hydrogen bonding units. Herein, an innovative self-healable PAT polymer containing plenty of thiourea hydrogen bonds was designed and introduced into FAPbI_3_ perovskite films (Figure 1). Significantly, abundant thiourea hydrogen bonds, endowing the PAT polymer with superior polymer transferability, afforded the improvement of the self-healing of cracks at GBs for damaged SPSCs. Accordingly, after 800 cycles of stretching, releasing and healing (at 100 °C for 15 min), the doped SPSCs retained 85% of their original IPCE. The healing characteristic were demonstrated in situ after stretching at 20% strain for 200 cycles. Moreover, pyridine units were also adapted to form strong intermolecular coordination interactions and passivate the grain boundary. The doped SPSCs prepared in 40% relative humidity (RH) demonstrated a champion efficiency of 19.58% with little hysteresis, almost rivalling those achieved in control atmosphere. This strategy of pyridine-based supramolecular doping in SPSCs paves a promising way for achieving efficient and self-healable crystalline semiconductors.

## 2. Experimental Section

### 2.1. Materials

Bis(3-aminopropyl) terminated poly(dimethylsiloxane) (PDMS-NH_2_, *M_n_* = 980 g/mol) were purchased from Gelest (Morrisville, PA, USA). Adipic dihydrazide (AD, 98%), 1,1′-thiocarbonyldiimidazole (TU), isophorone diisocyanate (IPDI), dimethyl formamide (DMF), and anhydrous tetrahydrofuran (THF, 99.5%) were purchased from Aladdin (Shanghai, China). Titanium aluminum carbide (Ti_3_AlC_2_, 200 mesh) and silver nanowires (AgNWs) were purchased from Beijing Kaifatetao Technology Co. Ltd. (Beijing, China). All the starting reagents and solvents used in the syntheses were used without further purification.

### 2.2. Synthesis of PAT Polymer

For the synthesis of PAT polymer, a THF solution (10 mL) of PDMS-NH_2_ (1 mmol) was stirred at 22 °C, into which a DMF solution (10 mL) of 1,1′-thiocarbonyldiimidazole (TU, 2 mmol) was dropped slowly. The solution was stirred at 22 °C for 48 h. Consequently, AD (1 mmol) as the chain extender, dissolved in DMAc, was added into the reaction system that was further kept at 40 °C for 15 h under stirring and N_2_ atmosphere. Viscous and transparent solutions of the PAT polymer were finally obtained. Then, the solution was slowly dropped into vigorously stirred MeOH (400 mL) to obtain the solid precipitate. ^1^H NMR (500 MHz, CDCl_3_, 25 ℃): δ (ppm) 1.26 (br, C*H*_2_), 1.45 (br, C(S)NHC*H*_2_ in octane), 3.49–3.53 (br, C(S)NHC*H*_2_), 7.29–7.54 (br, C(S)N*H*).

### 2.3. Fabrication of PSC Devices

Initially, both of the bottom and top transparent electrodes (hc-PEDOT:PSS) with a thickness of ~0.15 mm were prepared, as in previous reports [21]. Briefly, PEDOT:PSS (Heraeus CLEVIOSTM PH1000, Shanghai, China) with 20 mg/mL Zn(TFSI)_2_ (Sigma-Aldrich, Shanghai, China) were mixed together, then the ink were slot-die coated on the PDMS substrates via optimized shear stress. The conductivity is over 4000 S∙cm^−1^, which is comparable to that of the PET/ITO. As for matching the energy level alignment of device, a 30 nm hole-transport layer (HTL) PEDOT:PSS (Heraeus CLEVIOSTM Al4083, Shanghai, China) was then meniscus-coated the hc-PEDOT:PSS anode. Meanwhile, the PEI (Aldrich, Shanghai, China, 0.1 wt% diluted by isopropanol) was used to treat the PEDOT:PSS Al4083 layer for the top cathode. The 3M copper tape was applied to adhere to the electrode via silver glue.

The perovskite precursor with PAT dropping was prepared as below: 549 mg PbI_2_ (Sigma-Aldrich, Shanghai, China), 46 mg PbBr_2_ (Sigma-Aldrich, Shanghai, China), 150 mg HC(NH_2_)_2_I (Xi’an p-OLED Corp., Xian, China), 40 mg CH_3_NH_3_I (Xi’an p-OLED Corp.), PAT (0, 0.017 wt%, 0.035 wt% and 0.051 wt%) were dissolved in 1 mL mixture solvent of anhydrous DMF and anhydrous NMP (DMF:NMP, *v*/*v*, 9:1). The perovskite precursor solutions were spin-coated on the hc-PEDOT:PSS anode at a rotation speed of 4000 rpm for 30 s. Then, the perovskite films were heated at 100 °C on a hotplate for 15 min. Then, the perovskite precursor-coated substrate was annealed on a hot plate at 100 ℃ for 15 min. Subsequently, 60 nm-thick [6,6]-phenylC_61_-butyric acid methyl ester (PC_61_BM, ADS) was meniscus-coated from an anhydrous chlorobenzene solution. After drying, the top PEI/PEDOT:PSS/PDMS electrode was deposited via a film-transfer lamination technique: the bottom device was first exposed to air-plasma for about 2 s (flash) [22]. Then, PEI/PEDOT:PSS/PDMS electrode was transferred onto the PC_61_BM film and encapsulated using a vacuum-laminator [20]. All the processes were carried out in ambient environment with 40% relative humidity, room temperature.

### 2.4. Solar Cells Characterizations

The current density–voltage (J-V) curves are characterized using Keithley 2400 Source meter (Beijing, China,). The currents are measured under the solar simulator (EnliTech, Beijing, China, 100 mW cm^−2^, AM 1.5 G irradiation) and the reference silicon solar cell is corrected from NREL. All the measurements are performed under nitrogen at room temperature. The reverse scan range is from 1.3 V to 0 V and the forward scan range is 0 V to 1.3 V, with 8.0 mV for each step, and the scan rate is 0.2 V s^−1^, with a delay time of 30 ms. The incident photo-to-electron conversion efficiency spectra (IPCE) are detected under monochromatic illumination (Oriel Cornerstone 260 1/4 m monochromator equipped with Oriel 70613NS QTH lamp, Beijing, China,), and the calibration of the incident light is performed with a monocrystalline silicon diode. The area of PSCs was corrected by calibrated apertures (0.1 cm^2^). The repeated stretching cycle tests are performed by a custom-made stretching machine which is actuated by a stepper motor (Beijing, China, Zhongke J&M). All the results of stretching test results are averaged from over 50 samples. As for the self-healing J-V tests, all the samples were re-annealed on a hot plate at 100 ℃ for 15 min.

### 2.5. General Characterizations

X-ray diffraction patterns (XRD) were recorded with a Bruker D8 Discover Diffractometer (Beijing, China) with Cu Kα radiation (1.5406 Å). The step size, testing temperature and weight of the samples were 0.02°, 25 °C and 3 g, respectively. The testing angle (2θ), voltage and current were 5~80°, 40 kV and 40 mA, respectively. Top-view, cross-sectional SEM images were obtained with a field-emission scanning electron microscope (JEOL, JSM-7500F, Tokyo, Japan) at an accelerating voltage of 15.0 kV. AFM were obtained by using Veeco IIIa Multimode scanning probe microscope. The ultraviolet–visible (UV-Vis) spectra are recorded by Ocean Optics spectrophotometer (Shanghai, China). Steady-state photoluminescence (PL) and time-resolved photoluminescence (TRPL) measurements at the peak emission of ~770 nm (on the excitation at 470 nm) are carried out by the steady state and lifetime spectrometer (FLS920, Edinburgh Instruments Ltd. London, UK). The TRPL excitation fluence is ~4 nJ∙cm^−2^ from a 405 pulsed laser with a wavelength of 405 ± 8 nm and pulse width of 45 ps, at a repetition rate of 0.1 MHz. The PL decay data is recorded using time-correlated single photon counting technique. Fourier transform infrared spectra (FT-IR, Thermos Nicolet 6700 spectrometer, Berlin, Germany) were collected to characterize the surface chemical structure.

## 3. Results and Discussion

**Figure 2:**
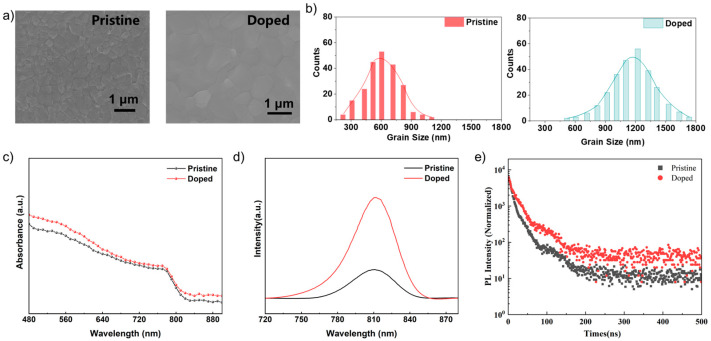
(**a**,**b**) Top-view SEM images and grain-size distributions of pristine and doped perovskites films. (**c**,**d**) The spectra of steady-state photoluminescence and optical absorption. (**e**) Fitted time-resolved photoluminescence decay spectra.

Various measurement techniques and theorical simulation were performed to verify the adequacy of PAT passivation crystallization upon the stretchable perovskite films. Initially, scanning electron microscope (SEM) of pristine and doped perovskite films were carried out to illustrate the enhancing of crystallinity (Figure 2a and Figure S5, Supporting Information). The grain size of perovskite crystals in SEM images were calculated by ImageJ software. ImageJ is a statistic analysis software based on Java designed by the National Institutes of Health (New York, NY, USA), which has been widely utilized to calculate and analyze the nanoparticle size in SEM images [13,20]. Obviously, with the incorporation of PAT, the grain-size enlarged remarkably from 0.650 μm to 1.25 μm (as shown in the inset graph). Furthermore, hydrophobic siloxane units were introduced into PAT molecular structure to increase the environmental stability. Contact angle (CA) measurements of pristine and doped perovskite films were carried out (Figure S4, Supporting Information). The CA increased from 62.59° to 78.32°, demonstrating that the siloxane structures in PAT result in an excellent hydrophobic characteristic, and the moisture-resistance of perovskite films was dramatically enhanced [23]. Subsequently, steady-state photoluminescence and optical absorption performances were explored and showed an apparent increase. As the optical absorption spectra illustrated, the utilization ratio of light spectrum markedly improved with the introducing of PAT polymer (Figure 2c). Furthermore, it could be found from fitted the time-resolved photoluminescence curves (TRPL, Figure 2d) that the perovskite films doped with PAT polymer displayed fast and slow phase lifetimes of τ_1_ = 8.6 and τ_2_ = 39.7 ns, respectively, whereas the pristine perovskite films exhibited lifetimes of τ_1_ = 5.3 ns and τ_2_ = 24.2 ns, respectively. Attributed to this decay, the concentration of trap-states tended to be lower and the electronic quality of doped films were improved intensively [16,24]. Additionally, XRD spectra were also patterned to explore the enhanced crystallinity as shown in Figure S6. The weak peak located at 14.4° and 28.2° can be indexed to the (110) and (220) plane of α-FAPbI_3_, respectively.

**Figure 3:**
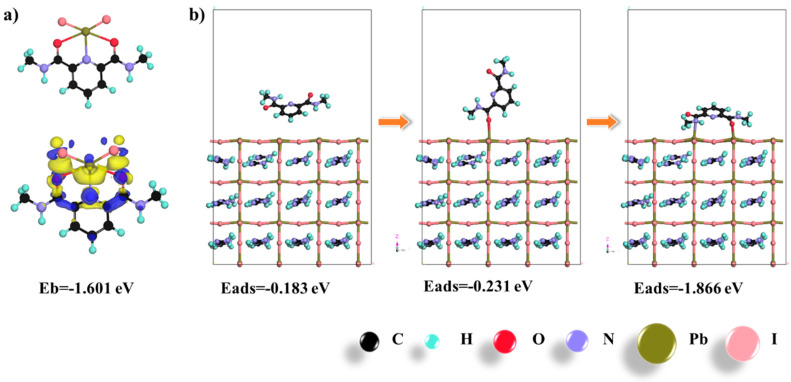
Theoretical models of chelation interactions between perovskites and pyridine units. (**a**) Absorption energy between PAT molecule and FAPbI_3_. (**b**) Different coordination states between PAT molecule and FAPbI_3_.

In theory, the compact coordination interactions between perovskite crystals and doped polymer could effectively passivate the GBs to withdraw various environmental stimuli. To profoundly demonstrate the mechanisms of improved crystallinity, first principle computational analysis based on density functional theory (DFT) was performed and the bichelation mechanisms were proposed. Briefly pyridine units in PAT, acted as Lewis bases and formed a chelation adduct with PbI_2_ (−1.601 eV) in the precursor, exhibiting strong intermolecular Pb^2+^-Namido, I^−^-Npyridyl, and Pb^2+^-Oamido coordination interactions (Figure 3a). Consequently, the higher energy barrier generated by the plentiful PAT-Pb^2+^ bichelation interactions would restrict the formation of FAPbI_3_ (formamidinium iodide as the organic cation). The higher energy barrier increased the critical concentration, contributing to the enlarged crystal grains. Moreover, the pyridine unit in PAT could be intensively adsorbed onto FAPbI_3_ surface with a higher adsorption-energy (−1.852 eV, Figure 3b) [25]. The original molecule and single-chelation passivation molecule showed an unstable state due to the lower adsorption energy, −0.183 eV and −0.231 eV, respectively. Additionally, iodine (I^−^) vacancy could also be passivated by pyridine units and compensated for the loss of electronic at GBs, preventing the photo-electrons being captured by these defects and thus reducing non-irradiative recombinations [26]. Therefore, attributed to these bichelation interactions of PAT as an inhibitor, intact crystallinity were achieved with oriented growth [27].

**Figure 4:**
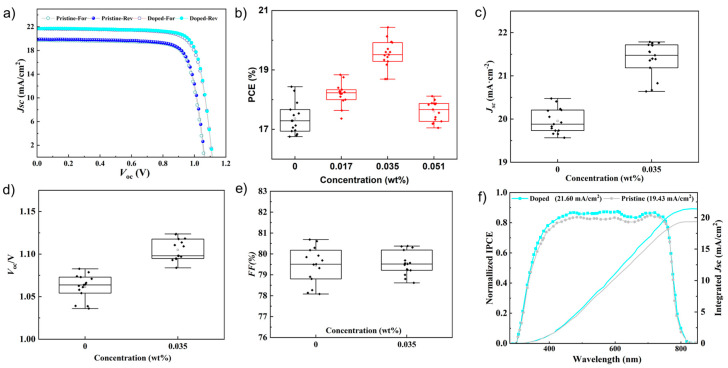
(**a**) J-V curves of the champion PSCs without and with PAT doping (Reverse directions noted as Rev; Forward directions noted as For; the effective area of devices is 0.09 cm^2^). (**b**–**e**) Power conversion efficiency (PCE), open-circuit voltage (*V*_OC_), short-circuit current (*J*_SC_) and fill factor (FF) of pristine and doped devices. (**f**) External quantum efficiency and integrated *J*_SC_.

To verify the feasibility of PAT dopant and the self-healing of photovoltaic devices, SPSCs with a structure of stretchable substrate (PDMS [28])/PEDOT:PSS/perovskites/PCBM/PEDOT: PSS/PDMS were assembled. To explore the optimal doping concentration of PAT, PCE of different SPSCs were tested as shown in Figure S7. It could be found that, with the increase in PAT concentration from 0 (0.05 mg∙mL^−1^) to 0.035 wt% (0.1 mg∙mL^−1^), PCE increased gradually from 17.51% to 19.58%, which could be attributed to the enhanced crystallinity. However, when the concentration of PAT increased to 0.051 wt% (0.15 mg∙mL^−1^), large amounts of pinholes formed in perovskite films, resulting in lower PCE. Based on these tests, 0.035 wt% (0.1 mg∙mL^−1^) concentration were selected as the optimal concentration. Then, the photocurrent density versus photovoltage (*J*-*V*) curves were tested and drawn in Figure 4a. The doped device exhibited a short-circuit current density (*J*_SC_) of 21.60 mA/cm^2^ and an open-circuit voltage (*V*_OC_) of 1.14 V under reverse-scan direction, a *J*_SC_ of 21.66 mA/cm^2^, a *V*_OC_ of 1.12 V under the forward-scan direction. Particularly, the champion PCE of 19.58% was firstly attained, which intrinsically verified the valid bichelation passivation by pyridine in PAT. Figure 4b–e display the detailed performance values of pristine and doped devices, demonstrating the excellent enhancing of SPSCs with PAT. The integration of the external quantum efficiency (EQE) spectra of the pristine and doped devices are shown in Figure 4f, being consistent with the data in Figure 4a.

It is well known that the moisture-induced volatilization of organic cations has been restricting the development of PSCs [12]. Subsequently, environmental stability, as well as photovoltaic performances, is also one of the fundamental challenges to be settled for SPSCs. The un-encapsulation-doped and pristine devices were fabricated as expected and exposed in atmosphere whose relative humidity (RH) was kept at around 20%. It is worth noting that the devices still remain 83% of original PCE after storing for 2000 h (Figure S8, Supporting Information). However, the PCE of pristine devices were only 65% of original devices. In addition to moisture, light stability was also verified under simulated solar light (AM1.5, 100 mW∙cm^−2^). As shown in Figure S8, even after continuously irradiating for 300 h, the doped SPSCs still kept almost 94.5% of their initial PCEs. On the contrary, the PCE of pristine devices decreased rapidly to 60% under the same conditions.

**Figure 5:**
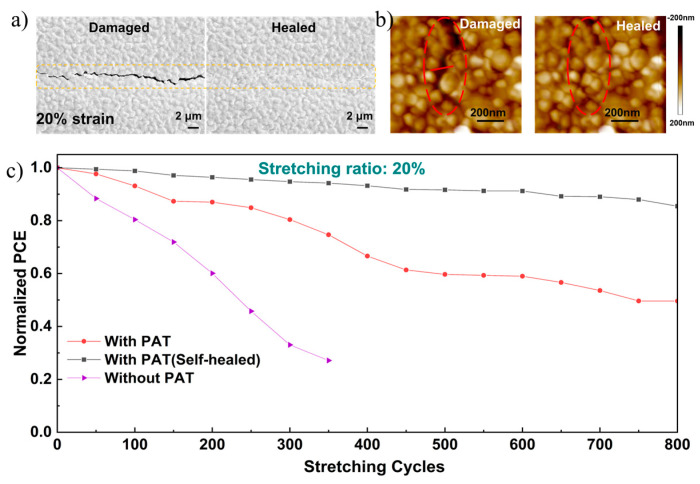
(**a**) SEM and (**b**) AFM images of damaged and healed perovskite films. (**c**) SPSC’s normalized average PCE as a function of stretching cycles with 20% strain.

Remarkably, the stretchability and self-healing were deeply explored to enrich the contents of PSCs to wearable and stretchable electronic devices [29,30,31]. The changes of perovskite films under 20% strain were investigated in ambient conditions, as shown in Figure 5a,b. Apparently, upon stretching to 20% strain, PAT at GBs containing plenty of thiourea hydrogen bonds were damaged firstly and dissipated the strain energy. After healing for 15 min at 100 °C, the little cracks mostly self-healed, verified in situ by SEM and AFM images performed. In addition, the self-healing of photovoltaic performances under 20% strain were also explored (Figure 5c) and verified for the doped SPSCs. Notably, even after 800 cycles of stretching and releasing, the doped SPSCs still retained 50% of their original IPCE, while the pristine ones retained 20%. However, after healing for 15 min, the IPCE of doped SPSCs dramatically increased to 85% due to the healing of cracks, demonstrating their excellent self-healing and stability. Compared with our previous work, the shorter healing time and higher healing efficiency may promote the practical applications of polymer-doped perovskite solar cells.

## 4. Conclusions

In summary, SPSCs with recoverable performances were successfully fabricated by introducing an innovative self-healable PAT with pyridine coordination units and plenty of thiourea hydrogen bonds. The doped SPSCs achieved a champion PCE of 19.58%, which is the best efficiency recorded to date for devices based on stretchable substrates. Moreover, moisture resistance and light irradiation resistance were also exhibited. Even after storing for 2000 h in 80% RH, the doped devices still retained 83% of their original PCE, attributed to the hydrophobic characteristic of siloxane in PAT polymer. Significantly, effective bichelation passivation and excellent self-healing properties were demonstrated by photovoltaic performances characterization and optical images performed in situ. After 800 cycles of stretching, releasing, and self-healing, the doped SPSCs retained 85% of their original IPCE. This strategy of bichelation passivation and thiourea hydrogen bonding healing offers a promising approach for crystalline semiconductors in wearable and stretchable electronic devices.

## Data Availability

The raw/processed data required to reproduce these findings cannot be shared at this time as the data also forms part of an ongoing study.

## Supplementary Materials

The following supporting information can be downloaded at: https://www.mdpi.com/article/10.3390/nano12183125/s1. Figure S1. Synthetic routes of the PAT polymers. Figure S2. Digital photographs showing the healing of the fractured PAT elastomer. (a) Orignal. (b) Cut sample. (c) Healed sample. (d) Stretched sample. Figure S3. Typical stress-strain curves of the intact, 10 min, 30 min and 1 h healed PAT elastomers at 25 ℃. Figure S4. The contact angle measurements. (a) Contact angle of Pristine perovskite films. (b) Contact angle of Doped perovskite films. Figure S5. Cross-section view of pristine and doped perovskite films. Figure S6. XRD spectrum of pristine and doped perovskite films. Figure S7. PCE tests of SPSCs with different PAT concentration. Figure S8. Stability tests of sealed cells. (a) Un-encapsulated cells with and without PAT with a RH level of approximately 80% and dark environment. (b) Under simulated solar light (AM 1.5; 100 mW/cm^2^).
