# Supplementary material for: Surface Chelation Enabled by Polymer-Doping for Self-Healable Perovskite Solar Cells
Source: Nanomaterials (Basel). 2022 Sep 9;12(18):3125. doi: 10.3390/nano12183125 (PMC9501477; doi:10.3390/nano12183125)
Supplement: Supplementary file 1 [file nanomaterials-12-03125-s001.zip › nanomaterials-1890271-supplementary.pdf]

nanomaterials

Supplementary Materials

Surface Chelation Enabled by Polymer-Doping for Self-Healable Perovskite Solar Cells

Kuiyuan Zhang [1,2,*], Xiangrong Shi [1], Guangyu Wu [3] and Yudong Huang [1]

1 MIIT Key Laboratory of Critical Materials Technology for New Energy Conversion and Storage, School of Chemistry and Chemical Engineering, Harbin Institute of Technology, Harbin 150001, China
2 School of Petrochemical Engineering, Changzhou University, Changzhou 213000, China
3 College of Biology and the Environment, Co-Innovation Center for the Sustainable Forestry in Southern China, Nanjing Forestry University, Nanjing 210037, China
* Correspondence: zky4995@hit.edu.cn

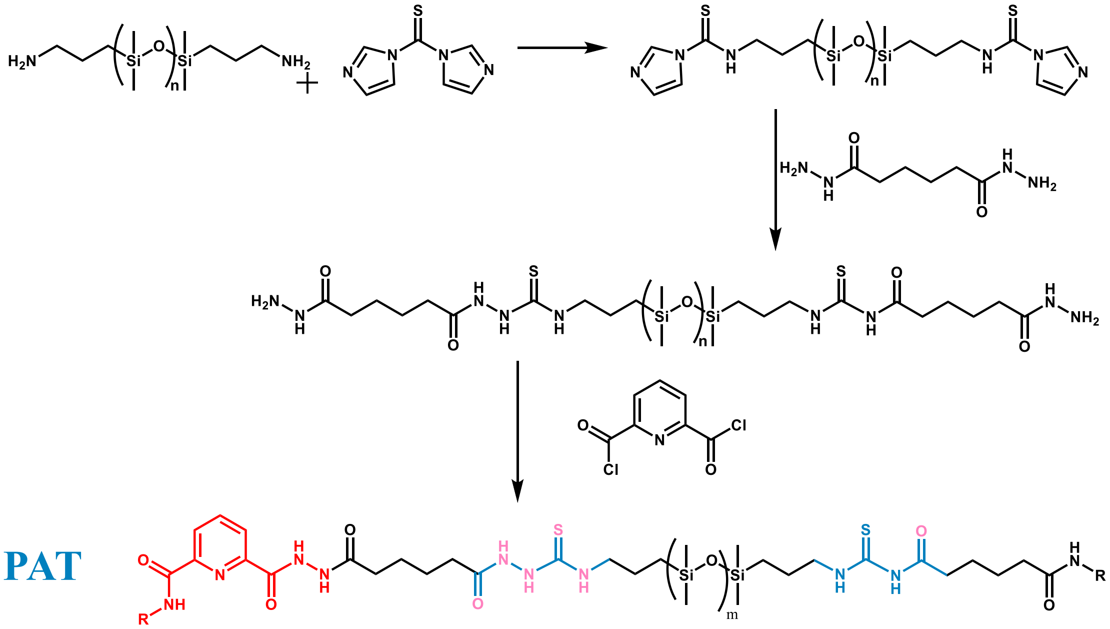

Figure S1. Synthetic routes of the PAT polymers.

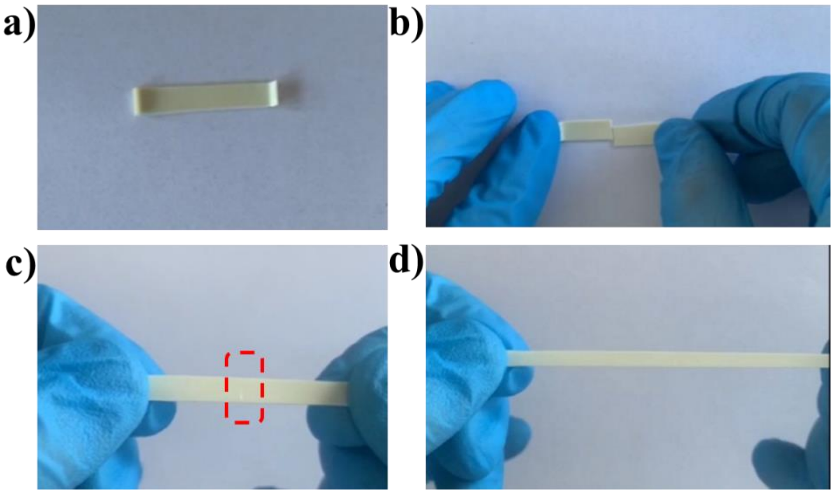

Figure S2. Digital photographs showing the healing of the fractured PAT elastomer. (**a**) Orignal. (**b**) Cut sample. (**c**) Healed sample. (**d**) Stretched sample.

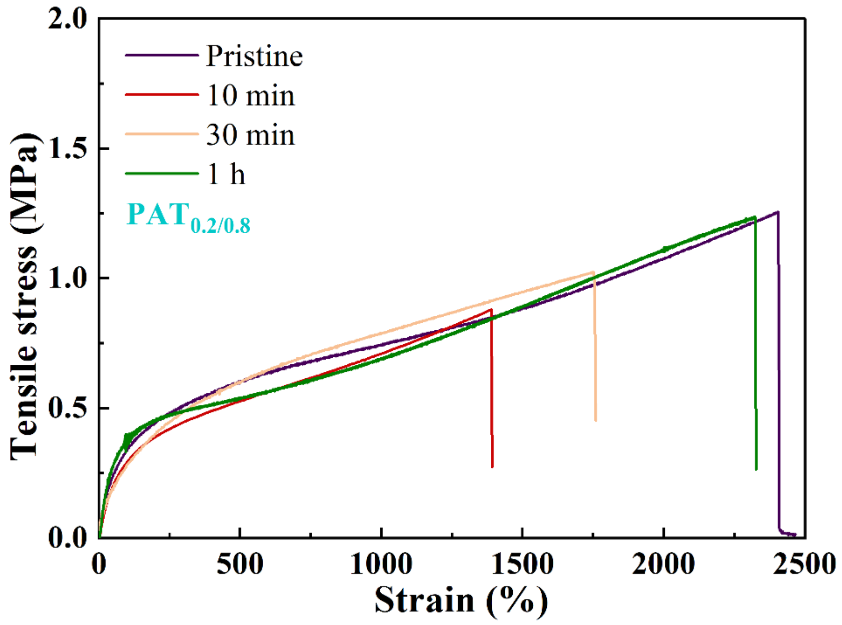

Figure S3. Typical stress-strain curves of the intact, 10 min, 30 min and 1 h healed PAT elastomers at 25 °C.

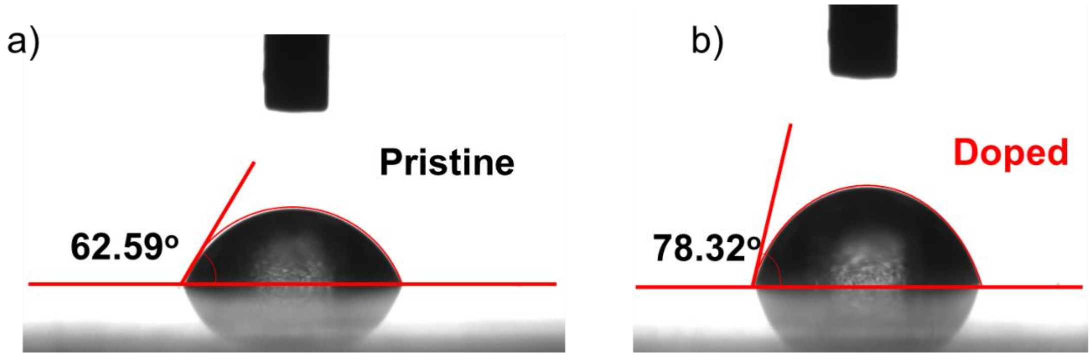

Figure S4. The contact angle measurements. (a) Contact angle of Pristine perovskite films. (b) Contact angle of Doped perovskite films.

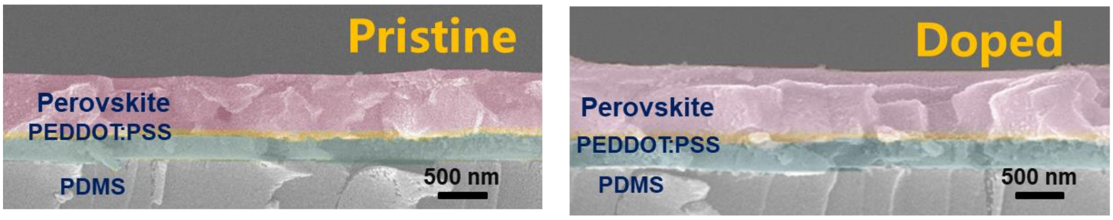

Figure S5. Cross-section view of pristine and doped perovskite films.

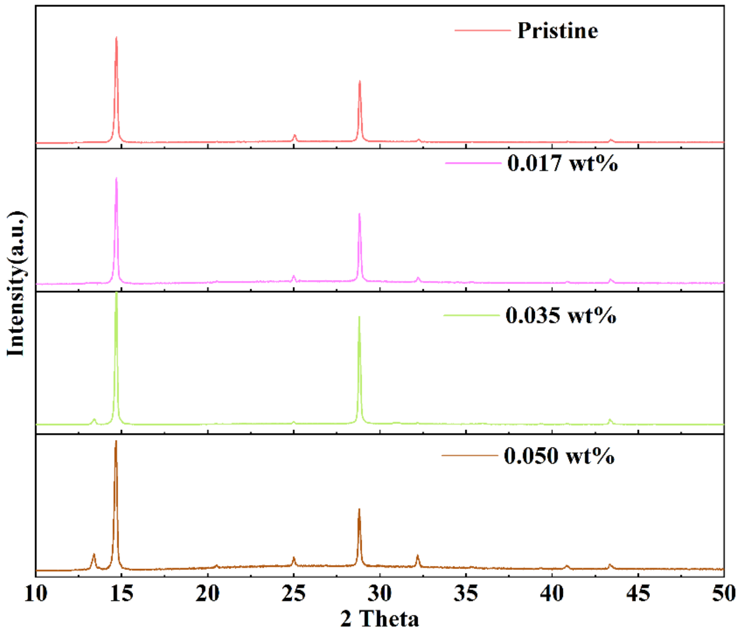

Figure S6. XRD spectrum of pristine and doped perovskite films.

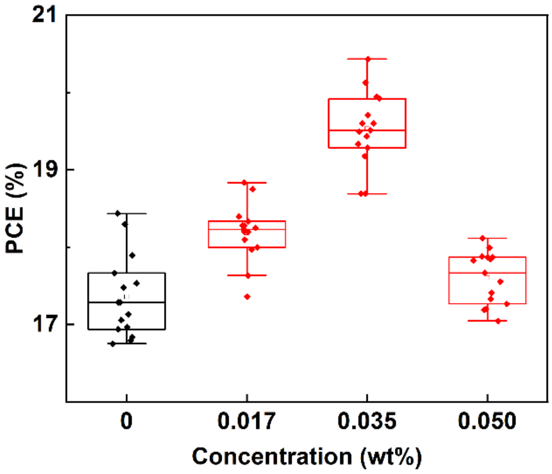

Figure S7. PCE tests of SPSCs with different PAT concentration.

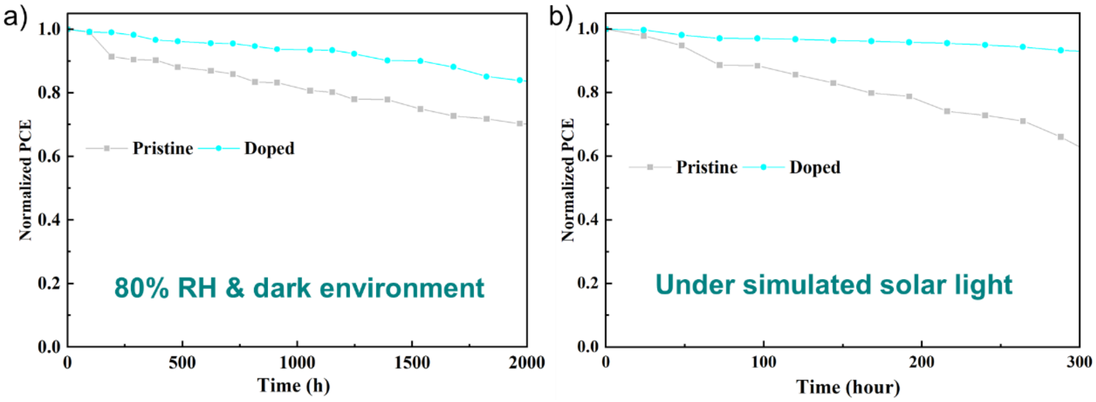

Figure S8. Stability tests of sealed cells. (**a**) Un-encapsulated cells with and without PAT with a RH level of approximately 80% and dark environment. (**b**) Under simulated solar light (AM 1.5; 100 mW/cm^2).
